# Reports of Cases

**Published:** 1899-02

**Authors:** 


### REPORTS OF CASES.

### A CASE OF PARALYSIS OF THE BOWELS.[1]

By James B. Rayner, V.S.,
WEST CHESTER, PA.

On March 24th I was called in to see a mare about six years old; found her very excitable, with two or three men on each side of her to hold her; found it very difficult for me to approach her; she would rear up and pitch headlong anywhere. Apparently blind at times, she would squat down as though she wanted to go under the trough.

I diagnosed it as a case of brain trouble, and it would be necessary to bleed her. The animal was so very nervous it was difficult to get near enough to use the lance, but after a time I succeeded.

The blood flowed very slowly, being very thick and dark in color.

I inquired of the owner how he had been feeding her. He replied, oats and corn. I also asked if she had any similar trouble heretofore. He said not any. He had purchased her only a few weeks ago from a party who had raised her. They were not able to break her, and turned her out to hunt her own living as best she could; did not feed her any grain at all. After the present owner had bought her he commenced to break her, and fed her regularly to get her in good condition. He drove her to and from West Chester daily, a distance of four miles; when driving would go very lively.

I considered, from the low condition she was in when he bought her, then feeding her heavily and driving, that it had brought on an impaction which produced paralysis of the bowels.

As it was utterly impossible to administer medicine in a liquid form, I concluded to try a strong purgative in the form of a ball, given three times a day, composed of aloes, one ounce; bromide of potassium, one-half ounce; powdered mandrake, two drachms; ground ginger, two drachms; croton oil, twenty-five drops. This had no effect whatever upon the bowels, which were completely paralyzed; but the brain responded very nicely to the bromide.

[1] Read before the Keystone Veterinary Medical Association.

Eyesight returned; would take notice of objects round about her. On the third day ate a little hay and could drink water fairly well, but would not take feed.

After I had emptied the rectum, which contained a very small amount, nothing of any account was passed afterward. During the night of the third day she died. I did not have the satisfaction of a post-mortem.

---

### AMPUTATION OF THE TAIL.[1]

By J. H. Wattles, M.D., D.V.S.,
KANSAS CITY, MO.

Amputation of the tail, while it is prohibited by law in many States, becomes a surgical necessity at times, and every practitioner should be able, with the instruments contained in an ordinary pocket-case, to perform every operation known to veterinarians without calling upon bystanders for assistance in controlling the animal during the operation, and especially in this one particular.

All that is necessary for this operation is one assistant to hold the twitch applied to the nose, an ordinary pair of side-lines put on in the usual manner and carried to the collar and fastened, to prevent the surgeon from being kicked by the animal, as the position taken is directly behind the horse.

After adjusting the side-lines and twitch, the hair of the tail above where the amputation is to be made is braided and tied firmly, then a circle of hair is cut off for a space of two or three inches, a stout piece of hempen twine is now wrapped several times around the tail about an inch above where the incision is to be made, and tied very tightly; this will stop all hemorrhage. The joint is felt for, and the one usually selected is about nine inches from the root of the tail. It can be easily found, as the tail bones are much larger at the articular extremities than in the bodies of the bones.

The tail is firmly grasped in the left hand, and with a very narrow-bladed bistoury, not to exceed a quarter of an inch in width, the incision is made through the joint selected from above downward. After the bones are disarticulated—and it should be done by the smallest possible incision—the knife is carried outward and toward the end of the tail, making a clean-cut flap, about two inches long, and the same process gone through with on the other side. This gives a V-shaped notch that can be closed perfectly,

[1] Read at the meeting of the Illinois Veterinary Medical and Surgical Association, Jan. 12, 1899.

making an interrupted suture of linen tape, and forming a perfect stump upon all parts of which hair will grow.

All the after-treatment necessary is to dust the line of sutures with a dry powder composed of oxide of zinc, powdered charcoal, with iodoform added, if indicated.

The ligature of twine can be gradually removed until there is no danger of excessive hemorrhage. It can be left on for an hour without danger or injury to the animal.

